# Comparative genomics of plant pathogenic *Botrytis* species with distinct host specificity

**DOI:** 10.1186/s12864-019-5580-x

**Published:** 2019-03-12

**Authors:** Claudio A. Valero-Jiménez, Javier Veloso, Martijn Staats, Jan A. L. van Kan

**Affiliations:** 1 Laboratory of Phytopathology, Wageningen University, 6708PB Wageningen, the Netherlands; 2 Department of Biology, Faculty of Sciences, University of A Coruña, A Coruña, Spain; 3 Biosystematics Group, Wageningen University, 6708PB Wageningen, the Netherlands; 4 Present address: RIKILT Wageningen University and Research, 6708WB Wageningen, the Netherlands

**Keywords:** Effector, Grey mould, Necrotroph, Secondary metabolite, Secretome

## Abstract

**Background:**

Fungi of the genus *Botrytis* (presently containing ~ 35 species) are able to infect more than 1400 different plant species and cause losses in a wide range of crops of economic importance. The best studied species is *B. cinerea*, which has a broad host range and is one of the best studied necrotrophic plant pathogenic fungi. Most other *Botrytis* spp. have a narrow host range and have been studied in less detail. To characterize genomic variation among different representatives of *Botrytis* spp., we sequenced and annotated the draft genomes of nine *Botrytis* species: *B. calthae, B. convoluta, B. elliptica, B. galanthina, B. hyacinthi, B. narcissicola, B. paeoniae, B. porri and B. tulipae.*

**Results:**

Bioinformatics and comparative genomics tools were applied to determine a core of 7668 shared protein families in all *Botrytis* species, which grouped them in two distinct phylogenetic clades. The secretome of all nine *Botrytis* spp. was similar in number (ranging from 716 to 784 predicted proteins). A detailed analysis of the molecular functions of the secretome revealed that shared activities were highly similar. Orthologs to effectors functionally studied in *B. cinerea* were also present in the other *Botrytis* species. A complex pattern of presence/absence of secondary metabolite biosynthetic key enzymes was observed.

**Conclusions:**

Comparative genomics of *Botrytis* show that overall, species share the main signatures and protein families in the secreted proteins, and of known effectors. Our study provides leads to study host range determinants in the genus *Botrytis* and provides a stepping stone to elucidate the roles of effector candidates in the infection process of these species.

**Electronic supplementary material:**

The online version of this article (10.1186/s12864-019-5580-x) contains supplementary material, which is available to authorized users.

## Background

The fungal genus *Botrytis* comprises ~ 35 species, which all interact with plants [1, 2]. With one exception, they are notorious pathogens with a necrotrophic infection behaviour, i.e. they kill host cells and invade the dead cells to acquire nutrients. The single exception, *B. deweyae*, can grow as a symptomless endophyte inside its host plant *Hemerocallis*, and occasionally switches lifestyle into a necrotizing infection destroying the young foliage, thereby causing ‘spring sickness’ [3]. The two most extensively studied species with a necrotrophic lifestyle, *B. cinerea* and *B. pseudocinerea*, are morphologically indistinguishable and cause grey mould on a broad range of (> 1400) host plant species [4]. Other *Botrytis* species are considered to be restricted to a single host species or a small number of taxonomically related hosts [5]. Phylogenetic analysis has separated the genus *Botrytis* into two distinct clades, one of which comprises *B. cinerea* and *B. pseudocinerea* along with a small number of species pathogenic on dicot plants, while the second (large) clade comprises mainly species pathogenic on monocot host plants, and especially on bulb-producing monocot species [1, 2, 5]. Many ornamental bulb flower crops of economic relevance (tulip, lily, Narcissus, snowdrop, Gladiolus) are each infected by a separate specialized *Botrytis* species [5].

Fungi with a necrotrophic lifestyle achieve plant cell death by the induction of an apoptotic programmed cell death in the host [6]. Rather than brutally killing the host cells, these pathogens co-opt the plant cell death machinery to their own benefit. Evidence is accumulating that many plant pathogenic fungi with a necrotrophic lifestyle secrete effector molecules (proteins and/or secondary metabolites) that trigger the host cell death machinery, following effector recognition by a cognate receptor protein in the host plant [7]. In interactions of *Parastagonospora nodorum* with its host (wheat), several cases have been described of inverse gene-for-gene interactions, in which a *P. nodorum* effector protein genetically interacts with a single genetic locus in the host that confers response to the effector and susceptibility to a fungal isolate producing this effector [8]. The cyclic non-ribosomal peptide victorin from *Cochliobolus victoriae* also triggers apoptotic programmed cell death in plants carrying the genetic locus for sensitivity [9, 10].

Several plant cell death-inducing proteins and metabolites were identified in *B. cinerea* [11–14], all of which act indiscriminately on all dicot plants that were tested. None of these necrotrophic effectors was essential for virulence of *B. cinerea* and none of them is considered to play a role in host specificity. Yet, there is evidence for the production of necrotrophic effectors in some specialised *Botrytis* species. The lily pathogen *B. elliptica* produced in liquid culture a proteinaceous effector activity that caused apoptotic cell death exclusively in lily, and conferred upon the non-pathogen *B. cinerea* the capacity to infect lily following effector infiltration [15]. Also the broad bean pathogen *B. fabae* was reported to produce a host-specific effector activity [16]. In order to understand the mechanisms of infection of host-specific *Botrytis* species, as compared to the broad host range pathogen *B. cinerea,* we generated draft genome sequences of nine *Botrytis* species, mostly selected from the phylogenetic clade of monocot-infecting pathogens, with emphasis on species infecting ornamental flower bulb crops. The genomes were analysed with special attention for the presence of genes that potentially contribute to the host specificity, such as genes encoding effector proteins, enzymes involved in secondary metabolite biosynthesis or enzymes involved in the degradation of monocot versus dicot cell wall polysaccharides.

## Results and discussion

### Sequencing and assembly

The genome assembly sizes of the nine *Botrytis* species ranged from 43 Mb to 55 Mb (Table 1). The genomes of eight species are similar in size to the previously described genome of *B. cinerea* (43.5 Mb; [17]), while the genome of *B. narcissicola* was ~ 10 Mb larger than the other species analysed here. Contigs that contained mitochondrial genes were identified with Blast and removed from the assemblies. To estimate the completeness of the assembled genomes, Benchmarking Universal Single-Copy Orthologs (BUSCO) was used. This analysis indicated that all genomes had a high level of completeness (96.3–99.2%), with the *B. elliptica* genome being the most complete. The assemblies are predicted to encode between 12,033 and 12,663 protein-coding genes.

**Table 1:** Assembly and gene prediction information of *Botrytis* spp. genomes

Species	Scaffolds	Assembly Size	Largest Scaffold	N50	BUSCO complete/partial	Predicted genes	Secretome size	% of secreted proteins
*B. calthae*	3985	47.56 Mb	293,700	56,053	97.5 (98.5)	12,492	745	5.96
*B. convoluta*	2054	45.40 Mb	436,056	97,955	98.7 (99.1)	12,532	752	6.00
*B. elliptica*	5594	47.68 Mb	230,072	36,976	99.2 (99.3)	12,663	762	6.02
*B. galanthina*	3422	43.97 Mb	354,402	65,043	98.9 (99.0)	12,575	784	6.23
*B. hyacinthi*	2509	43.91 Mb	532,180	115,520	99.0 (99.2)	12,197	752	6.17
*B. narcissicola*	8392	54.85 Mb	289,279	43,203	96.3 (98.1)	12,341	730	5.92
*B. paeoniae*	1833	46.36 Mb	901,367	125,168	99.0 (99.2)	12,138	746	6.15
*B. porri*	4737	43.11 Mb	181,158	35,774	97.4 (97.9)	12,033	716	5.95
*B. tulipae*	653	45.45 Mb	738,496	172,411	98.8 (99.0)	12,656	750	5.93

### Phylogenetics and phylogenomics

**Fig. 1:**
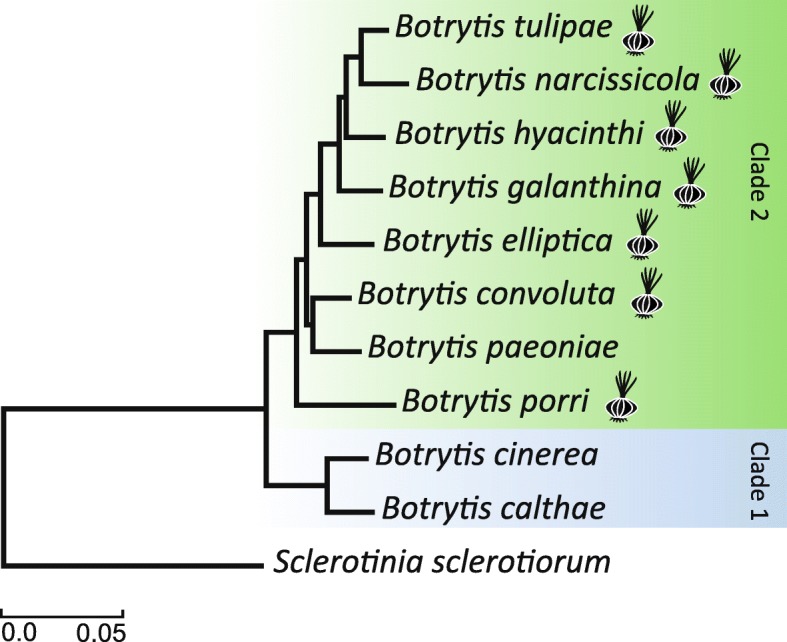
Phylogenetic tree based on single-copy orthologous genes of different *Botrytis* species, with *S. sclerotiorum* as the outgroup to root the tree. All branches have a high bootstrap support (ML > 90). Two clades previously reported in the genus *Botrytis* are highlighted. The bulb plant symbols next to the species names indicate species that infect monocotyledonous plants, species without the symbol infect dicotyledonous host plants

**Fig. 2:**
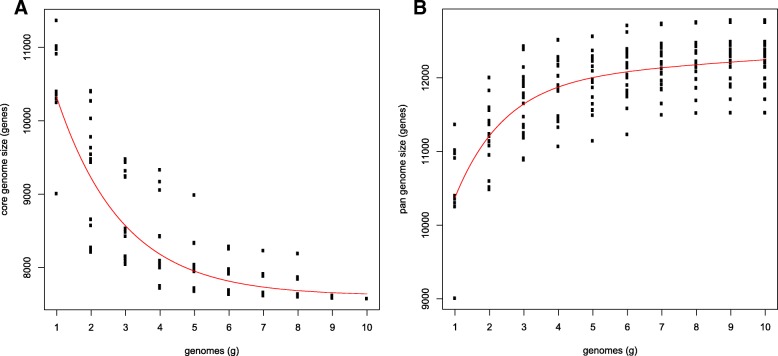
Pan-genome analysis of *Botrytis* spp. **a** Estimation of the *Botrytis* spp. core genome, in which the number of shared genes is plotted as a function of the number of species sequentially added. **b** Estimation of *Botrytis* spp. pan-genome size, in which the numbers of all genes are plotted as a function of the number of species sequentially added.

A phylogenetic tree was constructed of 7668 conserved core genes based on the amino acid alignment of ~ 3.7 million positions, and using *Sclerotinia sclerotiorum* as outgroup (Fig. 1). The relationship among the *Botrytis* species is in accordance with previous studies by [5], which divided the genus in two clades based on three protein-coding genes (G3PDH, HSP60 and RPB2). Clade 1 contains species that mainly infect eudicot plants, while species in Clade 2 infect either eudicot or monocot plants (but mainly monocots). A pan-genome analysis for the 9 *Botrytis* species sequenced in this study in combination with the previously sequenced *B. cinerea* B05.10 [17], indicated that the core genome of *Botrytis* spp. consists of 7617 orthologous gene clusters (Fig. 2a). On the other hand, the pan-genome consists of 12,245 orthologous gene clusters (Fig. 2b).

### GC content distribution

Previous studies have revealed that some fungal plant pathogens have distinct patterns in their genome, a so called two-speed genome, which consist of an alternation of repeat-rich and gene sparse regions with GC-equilibrated and gene dense regions [18, 19]. Moreover, it has been reported that repeat-rich regions can display an enrichment of rapidly evolving genes [20]. Analysis of the GC content in *Botrytis* spp. showed the presence of a bimodal distribution of GC content. The proportion of AT-rich regions in the genomes ranged from 4.86% in *B. cinerea* up to 27% in *B. narcissicola* (Table 2). The difference in genome sizes of the nine species, as compared to *B. cinerea*, is correlated with the proportion of AT-rich regions, as shown in *B. narcissicola*, the largest among the *Botrytis* spp. sequenced up to date (Table 2). Illumina-based sequence assemblies tend to lack repeats, especially AT-rich repeats [21]. Therefore it can be assumed that a more complete assembly of the nine genomes (based on long read technology) would have an even higher content of AT-rich repeats. Interestingly, *B. cinerea* has the lowest proportion of AT-rich regions in its genome even though it was sequenced with long read technology, which would encompass more difficult to assemble regions such as repetitive regions.

**Table 2:** GC content distribution among *Botrytis* spp.

	BCAL	BCIN	BCON	BELL	BGAL	BHYA	BNAR	BPAE	BPOR	BTUL
Genome Size	47.4	42.6	45.3	47.6	43.9	43.8	54.8	46.2	43	45.3
%AT rich regions	15.6	4.86	12.6	12.7	6.45	9.79	27	15.9	7.6	9.95
GC peak in AT rich regions	32.5	18.2	26.4	18.1	18.1	25	27	30.5	19.7	16.2
Genes in AT rich regions	99	0	8	4	1	6	17	33	4	2
Gene density in AT rich regions	13.3	0	1.4	0.66	0.35	1.4	1.15	4.49	1.22	0.44
Range of GC content in AT rich regions	0–37	0–27.4	0–33.6	0–28.6	0–27.7	0–32.6	0–35.4	0–36	0–28.7	0–27.5

The number of genes located in these AT-rich regions differed per species, with *B. calthae* having the highest number of genes (99) and *B. cinerea* the lowest (0). In order to elucidate if the AT-rich regions resulted from Repeat-Induced Point mutation (RIP), a meiosis-specific mechanism that affects duplicated sequence regions [22, 23], we compared the frequencies of dinucleotides between AT-rich regions and GC-equilibrated regions (Additional file 1). The AT-rich regions contained a strongly elevated frequency of TpA, which is the primary product of RIP in fungi, and is a strong indicator of RIP activity in these genomes [22]. Furthermore, RIP indices (ratio of TpA/ApT dinucleotide frequency) indicate RIP activity in all *Botrytis* spp. analysed here (Additional file 2).

### Secretome functions and effector proteins

**Fig. 3:**
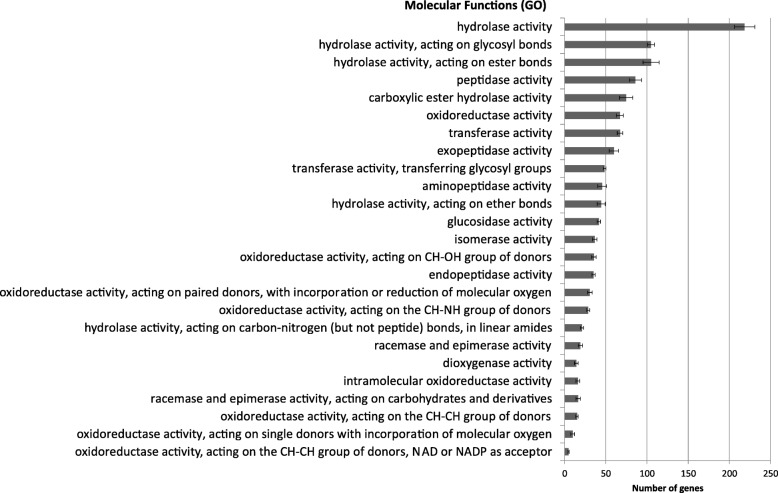
Number of genes encoding secreted proteins in *Botrytis* species grouped by GO annotation for the Molecular Function domain. The average of all species is shown. The error bars indicate the deviation in number of genes between the species

The secretome of all nine *Botrytis* spp. was similar in number (ranging from 716 proteins in *B. porri* to 784 in *B. galanthina*), representing 5 to 6% of the total proteome (Table 1), in line with what was reported for *B. cinerea* [17, 24]. The molecular function of the secreted proteins could be annotated for more than 50% of the secretome of each species (Fig. 3). There were no significant differences between the species in any of the Gene Ontology categories (error bars in Fig. 3 denote variability between species). Hydrolase activity (GO:0016787) was the most common molecular function of the secretome for all *Botrytis* species (around 25% of the total secretome), similar to what was reported previously for *B. cinerea* [25]. *B. galanthina*, *B. hyacinthi* and *B. cinerea* have the highest number of genes with hydrolase activity (approx. 230), while *B. porri* and *B. calthae* have the lowest number (approx. 200 genes). Half of the genes with hydrolase activity are plant cell wall degrading enzymes (PCWDEs, Table 3) with a potential role in host tissue decomposition and pathogenesis. Oxidoreductase activity (GO:0016491) was present in the secretome of all *Botrytis* species, representing 10% of the total secretome. This activity was more abundant in *B. galanthina*, *B. cinerea* and *B. tulipae* with approx. 70 proteins, while it was less abundant in *B. porri* (56 proteins). Transferase activity (GO:0016740) was present in similar levels as the oxidoreductase activity (10% of the total secretome) and *B. porri* was among the species with a higher number of proteins (approx. 70) along with *B. elliptica* and *B. cinerea*, while *B. convoluta* and *B. calthae* had a lower number of proteins with this activity (approx. 60). The final fairly abundant molecular function was isomerase activity (GO:0016853), accounting for 5% of the total secretome in all the species. The annotations of the secreted proteins in the GO Biological Process domain were mainly related to carbohydrate metabolic processes (GO:0005976, GO:0000272, GO:0044262), cell wall (GO:0071554) and pigment processes (GO:0042440), while annotations in the GO Cellular Component domain were mainly the cell periphery (GO:0071944) and cell wall (GO:0005618), as is expected from secreted proteins (Additional file 3). The 10 *Botrytis* species did not show significant differences in the number of genes in any of the categories of these GO domains.

**Table 3:** Comparison of plant cell wall degrading enzymes (PCWDEs) among *Botrytis* spp.

Species	Cellulose	Hemicellulose	Hemicellulose or pectin side chains	Pectin	Plant or Fungal CWDEs	Total
*B. calthae*	17	30	12	32	21	112
*B. cinerea*	20	34	13	38	27	132
*B. convoluta*	18	31	12	34	25	120
*B. elliptica*	19	36	11	35	22	123
*B. galanthina*	19	35	12	36	23	125
*B. hyacinthi*	20	34	13	35	23	125
*B. narcissicola*	20	31	11	28	21	111
*B. paeoniae*	19	31	12	32	22	116
*B. porri*	19	30	12	33	22	116
*B. tulipae*	18	32	11	29	22	112

**Fig. 4:**
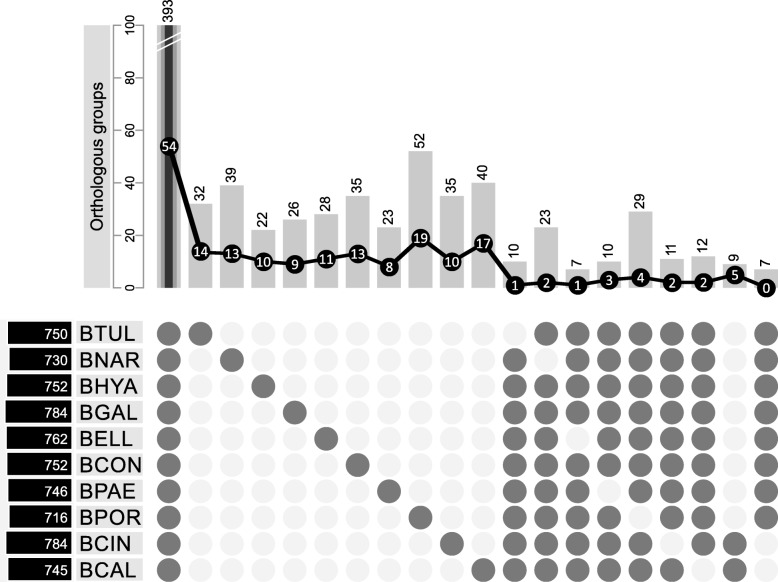
Presence/absence of orthologous groups of secreted proteins among *Botrytis* species. Species are listed in the order defined by the phylogeny in Fig. 1. Dark/white circles indicate the presence/absence of different subgroups in each species. The number of orthologous groups for each subgroup is shown above the bars. The total number of secreted proteins for each species is indicated in the left black box. The black line represents the number of predicted effectors per species in each subgroup. Two subgroups on the far right hand size represent proteins that are present only in species from Clade 1, or in species from Clade 2, respectively

All secreted proteins of the nine newly sequenced species and *B. cinerea* were clustered using Orthofinder and out of 7521 proteins, 7189 were assigned to 901 orthologous groups. A total of 393 orthologous groups were shared among all 10 *Botrytis* species (Fig. 4). Orthologs to known effectors or virulence factors from *B. cinerea* were present in this subset. For instance, the *Bcxyn*11A gene which encodes a necrosis-inducing xylanase [26], and *Bcpg*1 and *Bcpg*2, encoding endopolygalacturonases that are required for full virulence [27, 28], are present in all *Botrytis* spp. Furthermore, all *Botrytis* spp. contained an ortholog to the *Bcpls*1 gene, which encodes an integral membrane tetraspanin protein that participates in *B. cinerea* appressorium function and is required for full virulence [29]. Orthologs of the *Bcspl*1 gene, encoding a protein with a cerato-platanin domain that elicits plant defences and is required for full virulence [11] are also present in all *Botrytis* spp. The *nep*1 and *nep*2 genes, encoding phytotoxic effector proteins that induce necrosis and the synthesis of ethylene in plant leaves, are also present in all *Botrytis* spp., as previously reported [30, 31]. There are proteins from other plant pathogenic fungi that are involved in pathogenesis and contain a CFEM domain, which contains eight cysteines [32–34]. The mechanism by which CFEM domain-containing proteins act is unknown but a recent study reported that *B. cinerea* lacking *BcCFEM1* is less virulent [35]. Three orthogroups in the secretome contain a CFEM domain, of which the orthogroup of *BcCFEM1* is present in all *Botrytis* spp., another is shared between *B. calthae*, *B. cinerea*, *B. elliptica*, *B. hyacinthi*, *B. paeoniae* and *B. porri*, and the third orthogroup is only present in *B. porri*. Only the orthogroup shared between all *Botrytis* spp. is considered an effector based on EffectorP.

Besides the orthogroups shared by all ten *Botrytis* spp., an additional 332 orthologous groups are unique to only one single species (columns 2–11 in Fig. 4) and 102 groups (columns 12–18 in Fig. 4) are common to all species but one. Nine orthologous groups are present only in the two species from clade 1 (*B. cinerea* and *B. calthae*) and seven are present exclusively in the species from clade 2 (last two columns in Fig. 4) [5]. The nine orthologous groups unique to *B. cinerea* and *B. calthae* contain 5 candidate effectors of 9–15 kDa. Additionally, a hydrolase (hydrolyzing O-glycosyl compounds), a protein kinase and an oxidoreductase were present in orthologous groups unique to *B. cinerea* and *B. calthae*. The seven orthologous groups unique to the other eight species contain mainly hydrolytic enzymes (one serine-type peptidase (GO:0008236), one hydrolase acting on glycosyl bonds (GO:0004553), one asparaginase (GO:0004067) and one G1 endopeptidase (GO:0004190)) but no effectors.

In order to elucidate whether putative effector-encoding genes were clustered near repeats, and thus associated with rapidly evolving regions of the genome, we calculated the distances to the nearest repeat for all putative effector-encoding genes and compared them to the distances of a random subset of non-effector-encoding genes to the nearest repeat. Among the 10 species analysed, putative effector genes were on average significantly closer to repeats only in *B. cinerea* and *B. galanthina* but not in the other species (Additional file 4). The biological repercussion of this observation for *B. cinerea* and *B. galanthina* is unclear, however, there is no general tendency in *Botrytis* of proximity of effector genes to repeats, as was also observed in *S. sclerotiorum*, a close relative of *Botrytis* [36].

**Fig. 5:**
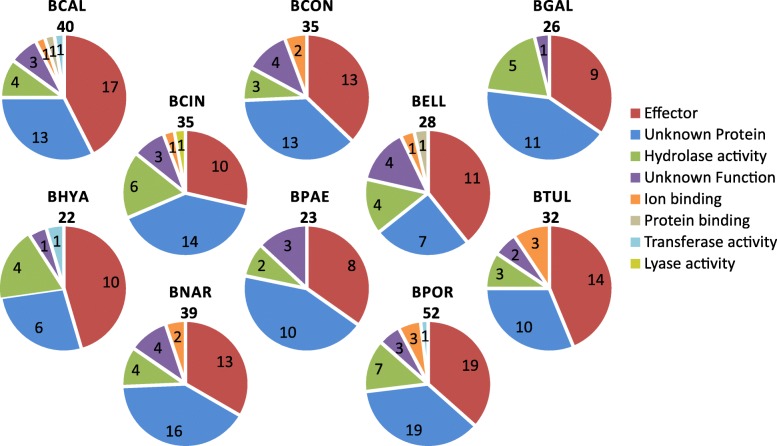
Unique proteins in the secretome for each *Botrytis* species. “Effector” proteins have been predicted with EffectorP. “Unknown protein” category refers to proteins for which no conserved domain has been found. “Unknown function” comprises proteins for which conserved domains have been found but no molecular function has been associated with the domain. The categories “Hydrolase activity”, “Ion binding”, “Protein binding”, “Transferase activity” and “Lyase activity” include proteins with a conserved domain associated with the GO molecular functions GO:0016787, GO:0043167, GO:0005515, GO:0016740, GO:0016829 or any of their subcategories, respectively

The orthologous groups of proteins that are unique for one of the *Botrytis* species are shown in columns 2–11 of Fig. 4 (for a complete list, see Additional file 5). The different GO molecular functions and effector predictions for these species-specific proteins are shown in Fig. 5. There are 8–19 unique effectors in each species. Whether such species-specific effectors serve as host range determinants requires further functional studies, including the analysis of gene expression during host plant infection, the construction of targeted knockout mutants and the use of effector proteins (produced in heterologous expression systems) in assays for host-specific cell death induction.

Furthermore, each of the 10 species has its own set of unique hydrolases, however, only few of these hydrolase activities are unique for a single species: only *B. cinerea* possesses a unique lyase and *B. calthae* possesses a unique transferase activity, for which orthologs are undetected in the other nine species. Among the secreted enzymes that are unique to single *Botrytis* species could be enzymes that detoxify antifungal secondary metabolites produced by host plants as defense against pathogens, collectively known as phytoanticipins or phytoalexins. In order to effectively colonize its host, a pathogenic fungus must be able to inactivate the antifungal compound. Such enzymatic inactivation can involve oxidation or hydrolysis. There is ample evidence for the capacity of several *Botrytis* species to detoxify host antifungal compounds: *B. cinerea* can degrade resveratrol [37] from grapes and α- tomatine [38] in tomato, *B. tulipae* degrades tulipalin [39] from tulips, and *B. fabae* degrades wyerone acid [40] in broad bean. The genes encoding such enzymes have not been identified with the exception of the *B. cinerea* laccase gene *Bclcc*2 [41]. For most *Botrytis* species, however, even the phytoalexins in their host plants remain to be chemically characterized, and it remains elusive which types of detoxifying enzymes could play a role in pathogenicity.

We further analysed a subset of the secretome that is related to the degradation of plant cell wall carbohydrates, as this is an important process during host plant infection. The genomes of the nine *Botrytis* spp. contain between 111 and 125 plant cell wall degrading enzymes (PCWDEs), slightly less than in *B. cinerea* (132; see Table 3). The PCWDEs can be further subdivided depending on the substrate that they degrade: cellulose, hemicellulose, and pectin. Overall, the number of secreted enzymes capable of degrading cellulose, hemicellulose and pectin is very similar among *Botrytis* spp., except for *B. narcissicola* and *B. tulipae*, where the number of pectin degrading enzymes is lower (28 and 29, respectively). The pectin content of plants can differ significantly, especially between monocot and dicot hosts [42]. Despite the fact that seven of the ten *Botrytis* spp. analysed here infect monocots, the content of PCWDE encoding genes does not differ. This may be correlated with the fact that the monocot hosts of these seven species are members of the Asparagales and Liliales, known to have a relatively high levels of pectin [42] as compared to Gramineae. The large number of genes encoding pectin degrading enzymes in the *Botrytis* species infecting bulb flower crops therefore should not be considered unusual.

### Secondary metabolite gene clusters

**Fig. 6:**
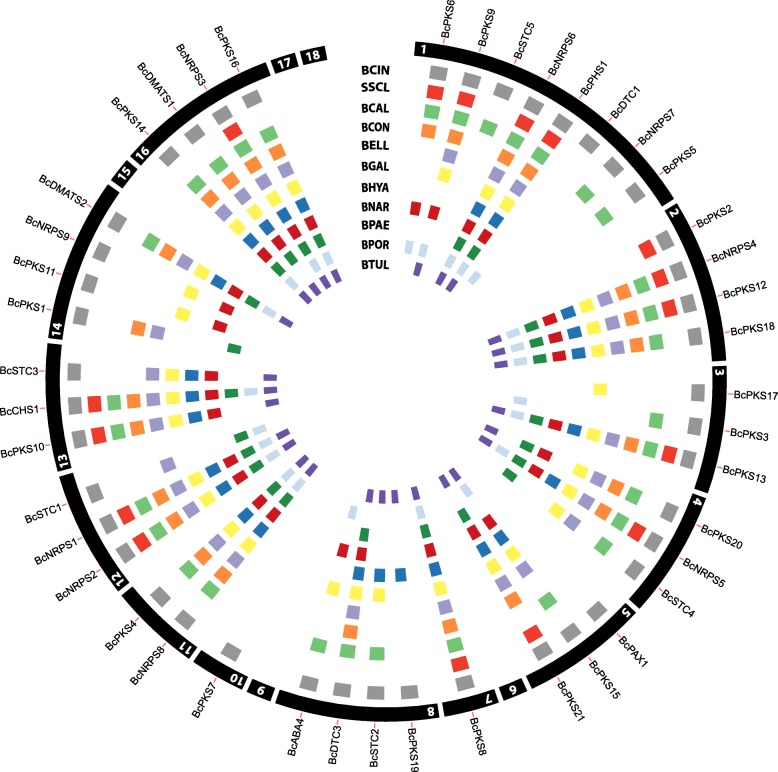
Visualization of the presence/absence of the secondary metabolite biosynthetic key enzymes of *B. cinerea* in nine other *Botrytis* species and *S. sclerotiorum*. The gene symbols for each key enzyme are along the perimeter of the outer circle depicting the 18 *B. cinerea* chromosomes (not to scale). The inner tracks with coloured blocks represent the presence of the secondary metabolite biosynthetic key enzymes for each species

Fungi are able to produce a wide array of compounds, defined as secondary metabolites (SM), that help them adapt and survive in different environments and compete with other organisms [43]. In *B. cinerea*, more than 40 secondary metabolite gene clusters have been identified, but only a few of the metabolites have been completely characterized, such as the well-characterized phytotoxins botrydial and botcinic acid (reviewed by [44]). The nine draft genomes were examined for the presence of secondary metabolite clusters present in *B. cinerea* by homology to the *B. cinerea* reference genome (Fig. 6)*.* The two key enzymes related to the production of melanin (Bcpks12 and Bcpks13), a key enzyme related to carotenoid synthesis, retinal (Bcphs1), and a key enzyme putatively involved in synthesis of pyrones, resorcylic acids and resorcinols (Bcchs1), are present in all nine species. Also key enzymes for the synthesis of coprogene siderophore (Bcnrps6) and other siderophores (Bcnrps2, Bcnrps3), that are found across all ascomycetes [45], are shared between *Botrytis* species. For other SM key enzyme-encoding genes, the distribution was heterogeneous. The key enzymes for the production of botcinic acid (Bcboa6/Bcpks6 and Bcboa9/Bcpks9) turned out to both be present in *B. calthae*, *B. convoluta*, *B. narcissicola* and *B. porri*, whereas *B. elliptica*, *B. galanthina* and *B. tulipae* only contained Bcboa9/Bcpks9 but not Bcboa6/Bcpks6. The SM key enzymes of the botcinic acid cluster were totally absent in *B. hyacinthi* and *B. paeoniae*. The key enzyme for botrydial synthesis (Bcbot2/Bcstc1) was present in *B. elliptica*, *B. paeoniae* and *B. porri* but not in the other species. The key enzyme for production of abscisic acid (Bcaba4) was present in *B. calthae*, *B. galanthina, B. narcissicola* and *B. porri* but not in other species.

The remainder of the *B. cinerea* key enzymes are involved in the synthesis of metabolites with an unknown function, but they can be classified based on the chemical nature of the metabolite synthesized by the gene cluster: peptides (non-ribosomal peptide synthetase), polyketides (polyketide synthase), terpenes (terpene synthase) and alkaloids (dimethylallyl tryptophane synthase). Of those, orthologs of some key enzymes are shared between all species (five polyketides, four peptides, and one alkaloid), some are only present in *B. cinerea* (indole-terpene Bcpax1 and peptide-polyketide Bcpks7), and for the rest of the key enzymes, the presence/absence varies per species (Additional file 6). In total, of the 43 key enzymes present in *B. cinerea*, *B. calthae* and *B. galanthina* shared the highest number of orthologs (31 key enzymes), while *B. paeoniae* and *B. porri* shared the lowest number (24 key enzymes) with *B. cinerea*, respectively. In total, there are 17 SM key enzymes that are present in all *Botrytis* species, which is noticeably low, when considering that there are 16 SM key enzymes shared between the more distantly related *S. sclerotiorum* and *B. cinerea* (Fig. 6). The SM key enzymes that are shared between *S. sclerotiorum* and *B. cinerea*, and *B. cinerea* and the rest of the *Botrytis* spp. are not the same, and only 11 key enzymes overlap between all *Botrytis* species and *S. sclerotiorum*.

The result of this comparison differs from a previous study which reported that 19 SM key enzymes were shared between *S. sclerotiorum* and *B. cinerea* [24]. This difference results from improvements of the assemblies and annotations in each species [17, 36]. For instance, the gene sizes of *pks*1 and *pks*18, which were previously reported to be shared between *S. sclerotiorum* and *B. cinerea*, have changed considerably, around 10% in the case of *Bcpks*1, and 64 and 300%, for *Bcpks*18 and *Sspks*18, respectively. Also, *Ssdmats*1 which had homology to *Bcdmats*1, is now more closely related to *Bcdmats*2. Interestingly, the updated annotation of *B. cinerea* B05.10 no longer contains a gene that encodes the diterpene cyclase *Bcdtc2*, which highlights the importance of having a good genome assembly and annotation*.*

The distribution of SM key enzyme genes among the ten *Botrytis* species analysed here is very patchy. The chemical structure of metabolites produced by most *Botrytis* SM gene clusters remain to be characterized, and there is no information under which conditions these gene clusters are expressed. Altogether, it is difficult to pinpoint SM gene clusters that may be interesting candidates for functional studies that aim to identify host range determinants.

## Conclusions

In this study we present the draft genome of nine species of *Botrytis* along with a comparative analysis with the previously sequenced *B. cinerea*. Our results show that overall *Botrytis* species share the main signatures and protein families in the secreted proteins. Furthermore, all effectors that were previously functionally characterized in *B. cinerea* are also present in other members of the genus. Analysis of the secondary metabolite biosynthetic gene clusters also gave insights in the complexity of presence/absence of these clusters. These genome data will provide leads to design hypotheses about candidate host range determinants in the *Botrytis* genus, either in the effector repertoire or in genes that participate in the tolerance to antimicrobial compounds of the host plant species. In the present study we especially focused on *Botrytis* species infecting ornamental flower bulb crops that are dispersed through the phylogeny of the species. The analysis of genomes of additional *Botrytis* species that are phylogenetically more closely related to each other will enable to zoom in on a further characterization of secreted proteins with potential roles in the infection process.

## Methods

### Strains and culture conditions

The fungal species used for this study are listed in Table 4. All fungal species were kept as conidial suspensions in 15% glycerol at − 80°C for long storage and grown on malt extract plates at 21°C before DNA extraction. DNA extraction was done as described by [46].

**Table 4:** Information about the strains used in this study

*Botrytis* sp.	Acronym	Isolate	Main host
*B. calthae*	BCAL	MUCL2830	*Caltha pallustris*
*B. cinerea*	BCIN	B05.10	> 1000 host species
*B. convoluta*	BCON	MUCL11595	*Iris*
*B. elliptica*	BELL	*Be9601*	*Lilium*
*B. galanthina*	BGAL	MUCL435	*Galanthus*
*B. hyacinthi*	BHYA	*Bh0001*	*Hyacinthus*
*B. narcissicola*	BNAR	M2120	*Narcissus*
*B. paeoniae*	BPAE	*Bp* 0003	*Paeonia* section *paeoniae*
*B. porri*	BPOR	MUCL3349	*Allium porrum*
*B. tulipae*	BTUL	*Bt9001*	*Tulipa*

### Sequencing and assembly

All sequences were sequenced with a paired-end library of 150 bp insert size, with a read length of 2 × 91 bp, except for *B. tulipae*, in which a paired-end library of 400 bp insert size, and an additional mate pair library of 3.5 kb were constructed. DNA from *B. tulipae* was sequenced by Macrogen (Korea) while DNA from the other eight species was sequenced by DNAVision (Liège, Belgium). De novo assembly was performed using the improved A5 pipeline [47], and further improved with Gapcloser from the Soapdenovo assembler [48]. For *B. tulipae*, assembly was performed using Spades assembler v.3.10.1 [49]. Completeness of the genome assemblies was assessed by the Benchmarking Universal Single-Copy Orthologs (BUSCO) v.2.0.1 software tool [50]. The GC content distribution of the sequenced genomes was examined using OcculterCut with default settings [19], and the analysis of repeat induced point mutations (RIP) was performed with RIPCAL [22].

### Genome annotation

The assembled genomes were annotated using the MAKER (v.2.31.9) pipeline [51]. Before annotation, a species-specific repeat library was constructed using RepeatModeler (v.1.0.8) in order to mask repeats [52]. Gene models were predicted with AUGUSTUS [53], SNAP [54], and GeneMark-ES [55] ab initio gene predictors. The gene models of the manually curated genome of *B. cinerea*, and all the fungal proteins available in the Swissprot database were provided as evidence for gene prediction. The predicted proteins were functionally annotated using BLASTp [56] against the non-redundant database of the National Center of Biotechnology Information (NCBI) and classified by InterProScan and Pfam analysis [57].

### Phylogenetic and phylogenomic analysis

The phylogenetic relationships were determined between all species sequenced, including the previously sequenced *B. cinerea* B05.10 [17], and using *S. sclerotiorum* [36] as the outgroup of the tree. The tree was constructed using 7668 single-copy orthologue genes, identified with Orthofinder [58]. The protein sequence for each gene was aligned and concatenated into a single matrix using MAFFT [59], and a maximum likehood phylogenetic tree was inferred with RAxML v.8.2.10 [60] using a generalized time reversible (GTR) plus GAMMA amino acid substitution model with 100 rapid bootstraps. A pan-genome analysis was done to calculate the number of core genes and was estimated using OrthoMCL [61] implemented in GET_HOMOLOGUES-EST [62] with e-value 1e^− 5^ and 75% coverage. For the pangenome analysis, only the orthogroups present in at least two species were included.

### Secretome, GOterm and effector prediction

Genes encoding putatively secreted proteins were identified for each *Botrytis* genome using several prediction tools. Signal-P v4.1 [63] was initially used to screen for a signal peptide, followed by TMHMM v.2.0 [64] to identify putative transmembrane domains. Proteins that did not have a signal peptide, or that had a transmembrane domain (a single transmembrane domain in the first 60 amino acids was allowed) were discarded. WoLF PSORT was used to predict protein localization [65]. Effectors were predicted using the EffectorP tool [66]. The GO enrichment in molecular functions was produced with the dcGO:database [67] and InterPro v66.0 [68]. To determine whether there was a relation between putative effector proteins and repeats, the distance to the nearest repeats was measured. Likewise, the distance between non-effector proteins and repeats was measured, using a random subset containing the same number of proteins as the set of effector proteins for that same species. The distances for the putative effector proteins were compared to the distances for the random non-effector proteins using Wilcoxon’s test in R (version 3.5.2).

### Secondary metabolite analysis

Identification of genes involved in the biosynthesis of secondary metabolites were based on homology to previously described genes in *Botrytis cinerea* and *S. sclerotiorum* [24, 44]. Sequences of the key enzymes were subjected to a BLASTn analysis to all *Botrytis* sp. genomes using an e-value of 1e^− 5^ and a sequence identity of 70%. Moreover, putative gene clusters that are predicted to be involved in biosynthesis of secondary metabolites were identified using antiSMASH (Antibiotics and Secondary Metabolite Analysis SHell) version 4.0.1 [69].

## Additional files

Additional file 1: Percentage differences between dinucleotides frequencies of AT-rich regions and GC-equilibrated regions. A positive number indicate a higher value in AT-rich regions and a negative number indicate a higher value in GC-equilibrated regions. (XLSX 11 kb)

Additional file 2: Fold differences of the dinucleotide frequencies of *Botrytis spp.* repeat elements relative to the control, and estimation of RIP indices. (PDF 316 kb)

Additional file 3: Number of genes encoding secreted proteins in *Botrytis* species grouped by GO annotation for the Biological Process and Cellular Component domains. The average of all species is shown, error bars indicate the deviation in number of genes between the species. (PDF 20 kb)

Additional file 4: Distance to nearest repeat region of putative effectors and a random gene set of non-effectors. The scale in the y axis is measured in bp to the nearest repeat. Asterisks represent different *P* values (* = *P* < 0.05; ** = *P* < 0.001; Wilcoxon’s test). (PDF 169 kb)

Additional file 5: List of putative effector genes present in *Botrytis* spp. (XLSX 28 kb)

Additional file 6: Summary of the presence/absence of all described secondary metabolites key enzymes of *Botrytis cinerea* in Botrytis spp. (XLSX 13 kb)

## Abbreviations

BUSCO: Benchmarking Universal Single-Copy Orthologs
G3PDH: Glyceraldehyde 3-phosphate dehydrogenase protein
GO: Gene Ontology
HSP60: Heat shock protein 60
PCWDE: Plant cell wall degrading enzyme
RIP: Repeat-Induced Point mutation
RPB2: RNA polymerase II protein
SM: Secondary Metabolites
