# Supplementary figures and images for: Comparative genomics of plant pathogenic Botrytis species with distinct host specificity
Source: BMC Genomics. 2019 Mar 12;20:203. doi: 10.1186/s12864-019-5580-x (PMC6417074; doi:10.1186/s12864-019-5580-x)

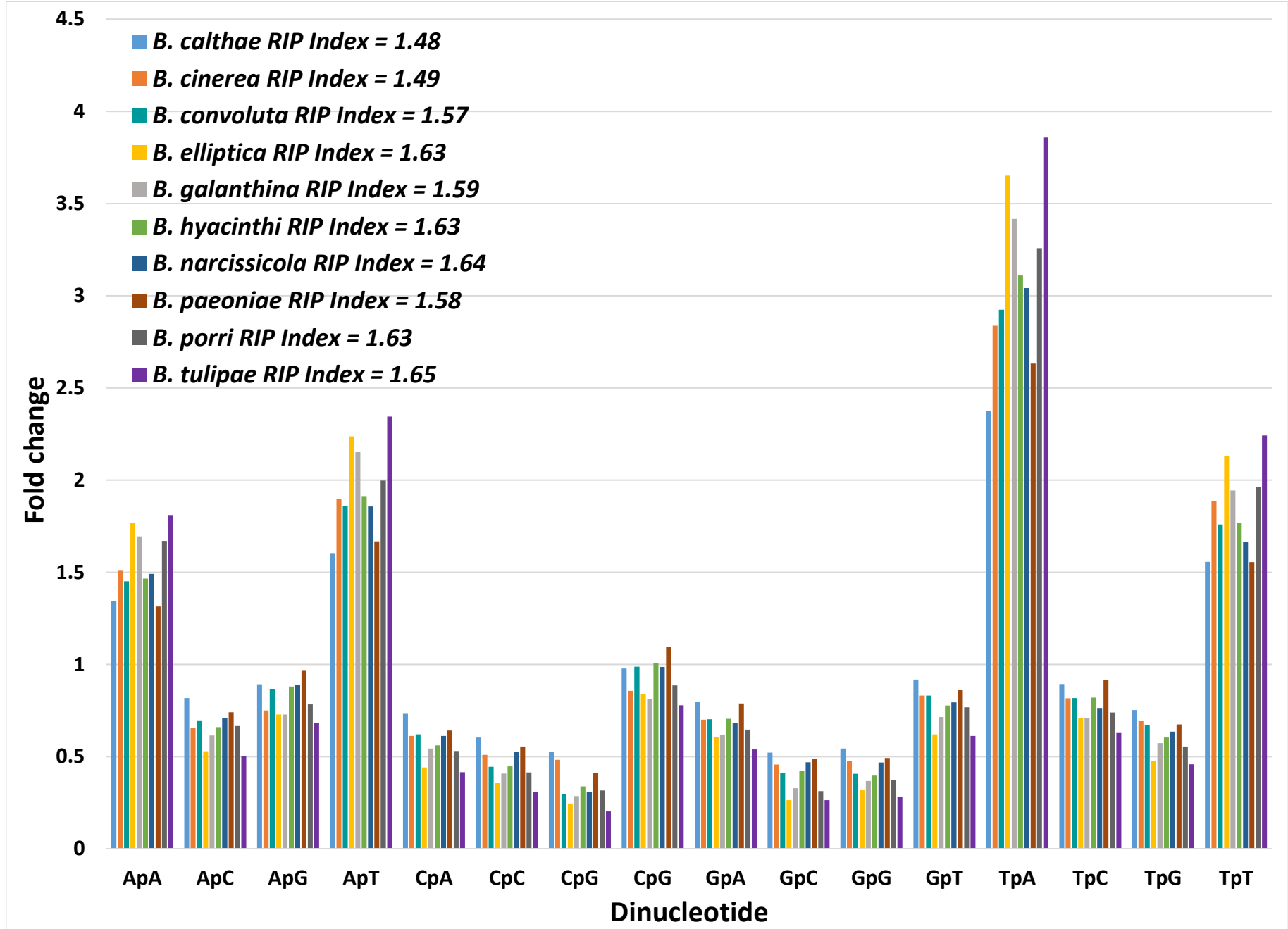

Supplement: Supplementary file 2 — Fold differences of the dinucleotide frequencies of Botrytis spp. repeat elements relative to the control, and estimation of RIP indices. (PDF 316 kb) [file 12864_2019_5580_MOESM2_ESM.pdf]

**Biological Process (GO)**

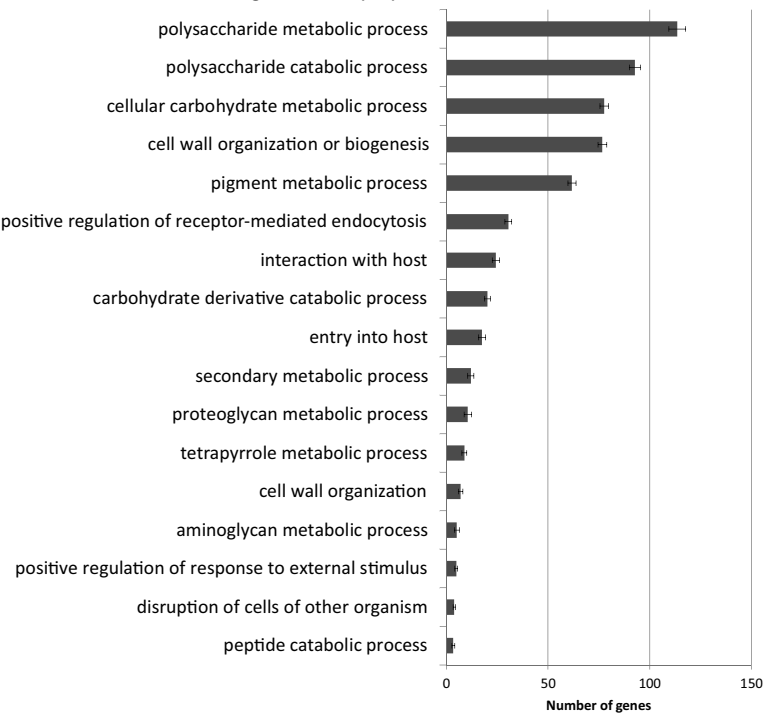

**Cellular Component (GO)**

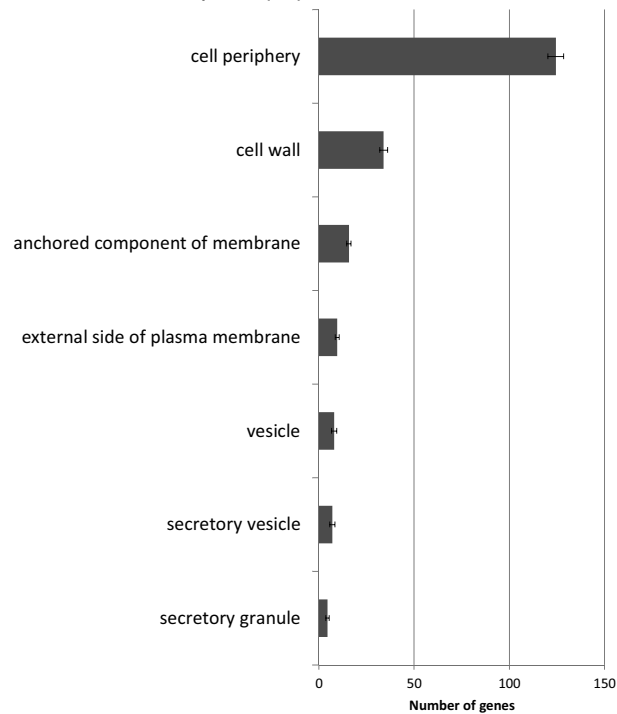

Supplement: Supplementary file 3 — Number of genes encoding secreted proteins in Botrytis species grouped by GO annotation for the Biological Process and Cellular Component domains. The average of all species is shown, error bars indicate the deviation in number of genes between the species. (PDF 20 kb) [file 12864_2019_5580_MOESM3_ESM.pdf]
