# Supplementary material for: Comparative genomics of plant pathogenic Botrytis species with distinct host specificity
Source: BMC Genomics. 2019 Mar 12;20:203. doi: 10.1186/s12864-019-5580-x (PMC6417074; doi:10.1186/s12864-019-5580-x)
Supplement: Supplementary file 4 — Distance to nearest repeat region of putative effectors and a random gene set of non-effectors. The scale in the y axis is measured in bp to the nearest repeat. Asterisks represent different P values (* = P < 0.05; ** = P < 0.001; Wilcoxon’s test). (PDF 169 kb) [file 12864_2019_5580_MOESM4_ESM.pdf]

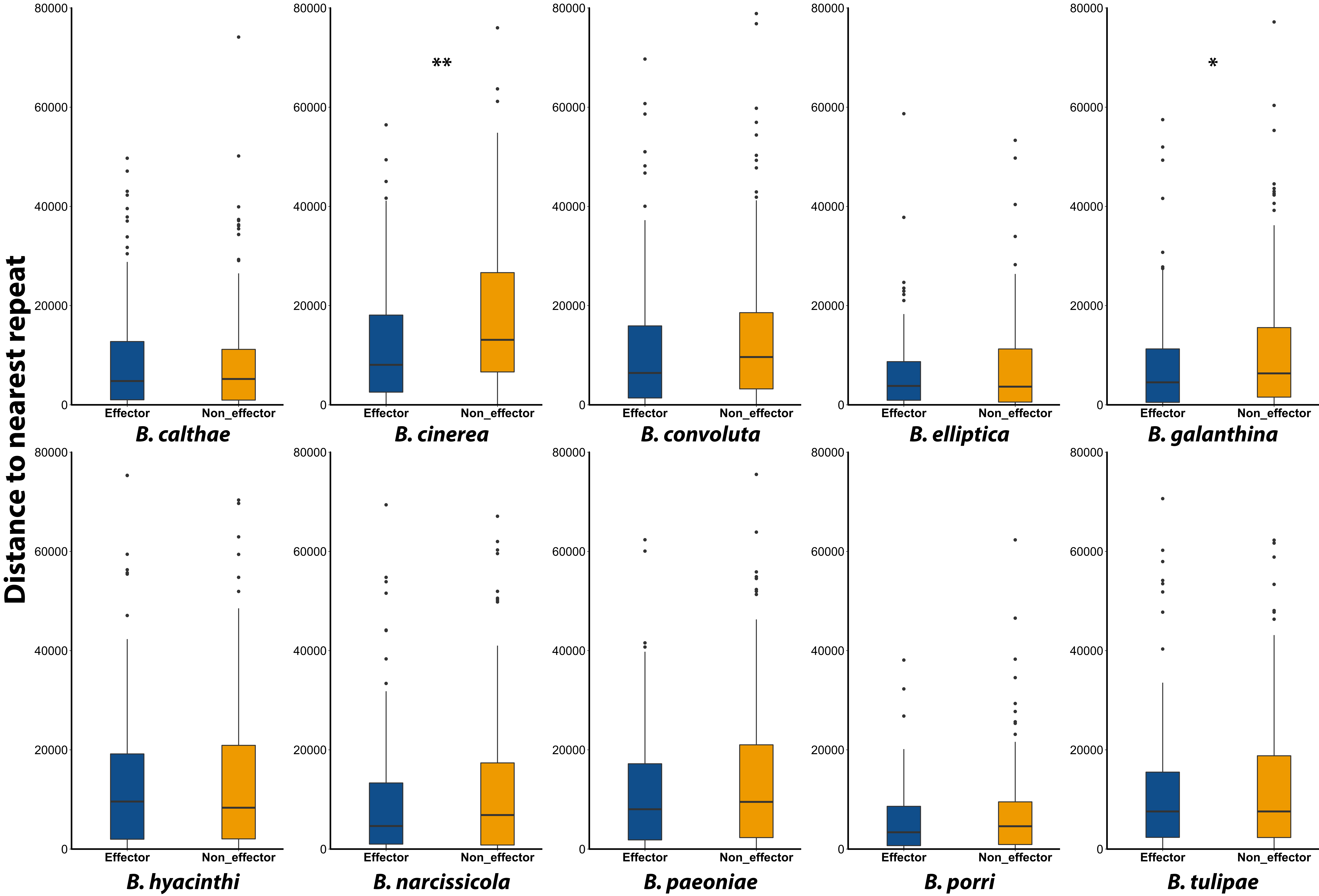

Distance to nearest repeat
80000
60000
40000
20000
0
Effector
Non_effector
B. calthae
**
B. cinerea
B. convoluta
B. elliptica
*
B. galanthina
B. hyacinthi
B. narcissicola
B. paeoniae
B. porri
B. tulipae
